# Predicting the risk of autoimmune thyroid disease in patients with vitiligo: Development and assessment of a new predictive nomogram

**DOI:** 10.3389/fendo.2023.1109925

**Published:** 2023-01-31

**Authors:** Ze Ma, Menghan Cai, Kang Yang, Junru Liu, Tao Guo, Xiaojie Liu, Junling Zhang

**Affiliations:** ^1^ Graduate school, Tianjin University of Traditional Chinese Medicine, Tianjin, China; ^2^ Dermatology and Medical Cosmetology Department, Laishan branch of Yantai Yuhuangding Hopitali, Shandong, China; ^3^ Department of Dermatology, Tianjin Academy of Traditional Chinese Medicine Affiliated Hospital, Tianjin, China

**Keywords:** vitiligo, autoimmune thyroid disease, nomogram, predictive model, lasso

## Abstract

**Background:**

This study aimed to develop an autoimmune thyroid disease (AITD) risk prediction model for patients with vitiligo based on readily available characteristics.

**Methods:**

A retrospective analysis was conducted on the clinical characteristics, demographics, skin lesions, and laboratory test results of patients with vitiligo. To develop a model to predict the risk of AITD, the Least Absolute Shrinkage and Selection Operator (LASSO) method was used to optimize feature selection, and logistic regression analysis was used to select further features. The C-index, Hosmer–Lemeshow test, and decision curve analysis were used to evaluate the calibration, discrimination ability and clinical utility of the model. Internally, the model was verified using bootstrapping; externally, two independent cohorts were used to confirm model accuracy.

**Results:**

Sex, vitiligo type, family history of AITD, family history of other autoimmune disease, thyroid nodules or tumors, negative emotions, skin involvement exceeding 5% of body surface area, and positive immune serology (IgA, IgG, IgM, C3, and C4) were predictors of AITD in the prediction nomogram. The model showed good calibration and discrimination (C-index: 0.746; 95% confidence interval: 0.701–0.792). The accuracy of this predictive model was 74.6%.In both internal validation (a C-index of 1000 times) and external validation, the C-index outperformed (0.732, 0.869, and 0.777). The decision curve showed that the AITD nomogram had a good guiding role in clinical practice.

**Conclusion:**

The novel AITD nomogram effectively evaluated the risk of AITD in patients with vitiligo.

## 1 Introduction

Vitiligo is a depigmenting skin disease that is prevalent in the population (1). The cause is currently unknown, but the most widely accepted theory is an autoimmune etiology; this has been supported by epidemiological, clinical, and experimental investigations (2–10). Epidemiologic reports have also confirmed that vitiligo is present in the autoimmune polyendocrine syndrome (11). Autoimmune thyroid disease (AITD) presents primarily as two clinical manifestations: Hashimoto’s thyroiditis and Graves’ disease (12). The prevalence of AITD in patients with vitiligo is 14.3% (13); in contrast, the prevalence of vitiligo in patients with AITD ranges from 2.7%–6.8% (14, 15). Currently, there is controversy regarding the order in which vitiligo and AITD arise, although some studies have suggested that vitiligo appears earlier (16–19). Others have reported that the probability of thyroid disease in patients with vitiligo increases with age (17). At this time, there is no clinical treatment for the source of AITD; in addition, prolonged AITD leads to serious and life-threatening complications (20). Therefore, there is a need to develop a model to identify, diagnose, and treat the damage caused by AITD at an early stage of the disease, and to predict whether a person will eventually experience AITD.

Cumulative studies have reported many factors closely related to the pathogenesis of AITD. A large proportion of women experience AITD; this may be owing to an important role of estrogen in the pathogenesis of AITD (21). In addition, the onset of AITD has often shown familial clustering and thus, common causative factors are under consideration (22). Patients with vitiligo have also been found more likely to have AITD when their immunoglobulin plus complement tests (IgA, IgG, IgM, C3, and C4) were abnormal, when they had thyroid nodules or tumors, and also when the Positive and Negative Affect Schedule (PANAS) assessment registered negative emotions (12, 23, 24). Another study has reported that different types of vitiligo have varying probability of developing AITD; this may be related to different clinical manifestations and pathogenesis (25). Patients with non-segmental vitiligo (NSV) are more likely to develop AITD.

This study aimed to provide an accurate and straightforward model to predict the presence of AITD in patients with vitiligo, using features and clinical symptoms that are simple for patients to understand. This predictive model can be helpful for the education and individualized treatment of patients with high-risk AITD; it might also reduce the burden of medical insurance and thus, ease communication and exchange of patients. In addition, this study and model might help to avoid the problem of overtreatment in the large population of vitiligo patients in China with varying socioeconomic status.

## 2 Materials and methods

### 2.1 Patients

We collected data from patients visiting the outpatient dermatology department at the Affiliated Hospital of the Tianjin Academy of Traditional Chinese Medicine between January 2019 and December 2020. Data collected from the same department between January 2021 and May 2022 were considered as external verification (Group 1). Patient data collected between January and December 2021 at the Yantai YuHuangDing Hospital were considered as external validation (Group 2). All patients had completed a detailed survey within the medical record including demographic characteristics, family history of vitiligo and AITD, history of smoking and drinking, and other basic AITD risk factor information. Follow-up data were collected at return visits and through telephone surveys. Two dermatologists made the diagnosis of vitiligo based on imaging results, such as skin confocal laser scanning microscopy, Wood’s lamp, or dermoscopy. Physicians and dermatologists diagnosed AITD based on clinical observations and laboratory tests. This study was approved by the Ethics Committee of the Affiliated Hospital of Tianjin Academy of Traditional Chinese Medicine, Tianjin, China. This was a retrospective study, approved by the Ethics Committee, and the need for informed consent was waived.

### 2.2 Evaluation criteria

Clinical vitiligo can be classified as segmental (SV), NSV, and mixed vitiligo (26). Typical SV displays a unilateral distribution, while NSV presents with bilaterally symmetrical white macules (24). The PANAS (27) emotional scale was used to evaluate the current emotional state of patients. Laboratory examinations assessed positive immunoglobulin and complement (IgA, IgG, IgM, C3, and C4) levels. Whether the area of vitiligo exceeded 5% of the entire body area was assessed according to the area of the palm of the hand. Unilateral palm area is approximately equal to 1%.The remaining parameters were obtained through patient interview or case review. Most indicators mentioned in this article were for the etiological determination and data retrieval required for vitiligo patients and did not add additional financial burden to the patient.

### 2.3 Statistical analysis

All information is reported as count (%), including demographic details, laboratory results, clinical symptoms, and vitiligo phases. SPSS (version 26.0; http://www.spss.com.cn), STATA (version 15.0; https://www.stata.com), and R (version 3.6.3; https://www.R-project.org) software were used for statistical analyses. All the above factors were included within the Least Absolute Shrinkage and Selection Operator (LASSO) method to reduce the dimensionality of the data (28). These were appropriate for choosing the top AITD risk-predicting features. In the LASSO regression model (29), we selected 11 features with non-zero coefficients, and statistical software was inputted to establish the prediction model using multivariate and univariate logistic regression analyses. We used odds ratios (ORs) and p-values with 95% confidence interval (CI). A predictive model for AITD was developed using the variables with a univariate p-value < 0.05 and a multivariate p-value in the logistic regression analysis of the optimal model. The Hosmer–Lemeshow test was used to evaluate calibration of the AITD nomogram. The C-index was calculated to measure the AITD nomogram’s discrimination performance. To measure the predictive power of the nomogram, the area under the curve (AUC) was plotted. The AITD nomograms were validated by bootstrapping (1000 bootstrapping resampling) to calculate the relative corrected C-index (30). To determine the C-index, two different queues (queues 1 and 2) were used to validate external validation. By measuring the net rate of return under various threshold probabilities using a decision curve analysis (DCA), the clinical value of the AITD nomogram was evaluated (31).

## 3 Results

### 3.1 Features of vitiligo and autoimmune thyroid disease

In this retrospective study, we reviewed the data of 703 patients with vitiligo, of whom 239 had concomitant AITD. All patient data, including demographic characteristics, laboratory tests, clinical manifestations, and stages of vitiligo and other information, are shown in **Table 1**. Regarding external validation, the Group 1 data were from 156 patients with vitiligo, of whom 55 had concomitant AITD. The Group 2 data comprised that of 42 patients with vitiligo and 14 with concomitant AITD.

**Table 1:** Patient-related characteristics and data.

	AITD (No)	AITD (Yes)	Total
Sex			
Female	238 (51%)	165 (69%)	403 (57%)
Male	227 (49%)	73 (31%)	300 (43%)
Age of vitiligo onset (years)			
≤15	12 (3%)	6 (3%)	18 (2%)
>15, ≤30	391 (84%)	214 (89%)	605 (86%)
>30, ≤45	48 (10%)	12 (5%)	60 (9%)
>45	14 (3%)	6 (3%)	20 (3%)
Duration (years)			
≤5	308 (66%)	169 (71%)	477 (68%)
>5, ≤10	137 (29%)	60 (25%)	197 (28%)
>10	20 (4%)	9 (4%)	29 (4%)
Vitiligo type			
MV	57 (12%)	7 (3%)	64 (9%)
NSV	284 (61%)	197 (83%)	481 (68%)
SV	124 (27%)	34 (14%)	158(23%)
Family vitiligo history			
No	422 (91%)	217 (91%)	639 (91%)
Yes	43 (9%)	21 (9%)	64 (9%)
Family AITD history			
No	452 (97%)	210 (88%)	662 (94%)
Yes	13 (3%)	28 (12%)	41 (6%)
History of ADs except thyroid			
No	409 (88%)	198 (83%)	607 (86%)
Yes	56 (12%)	40 (17%)	96 (14%)
Family history of ADs except thyroid			
No	451 (97%)	209 (88%)	660 (94%)
Yes	14 (3%)	29 (12%)	43 (6%)
Thyroid nodules or tumors			
No	444(95%)	208 (87%)	652 (93%)
Yes	21 (5%)	30 (13%)	51 (7%)
Trace element			
Normal	387 (83%)	192 (81%)	579 (82%)
Abnormal	78 (17%)	46 (19%)	124 (18%)
Mood			
Positive	375 (81%)	124 (52%)	499 (71%)
Negative	90 (19%)	114 (48%)	204 (29%)
Hypertension			
No	459 (99%)	235 (99%)	694 (99%)
Yes	6 (1%)	3 (1%)	6 (1%)
Hyperlipidemia			
Normal	459 (99%)	236 (99%)	695 (99%)
Abnormal	6 (1%)	2 (1%)	8 (1%)
Blood sugar status			
Normal	461 (99%)	233 (98%)	694 (99%)
Abnormal	4 (1%)	5 (2%)	9 (1%)
Cigarette history			
No smoking	413 (90%)	203 (85%)	616 (88%)
Smoking	32 (6%)	25 (11%)	57 (8%)
Quit smoking >6 months	20 (4%)	10 (4%)	30 (4%)
Alcohol use			
No drinking	403 (87%)	206 (87%)	609 (87%)
Quit drinking >1 year	32 (7%)	11 (5%)	42 (6%)
Drinking <3 times per week	14 (3%)	12 (5%)	26 (4%)
Drinking >4 times per week	16 (3%)	9 (3%)	25 (4%)
Area of involvement			
≤5%	445 (96%)	203 (85%)	648 (92%)
>5%	20 (4%)	35 (15%)	55 (8%)
Immune serology			
normal	415 (89%)	189 (79%)	604 (87%)
abnormal	50 (11%)	49 (21%)	94 (13%)

AITD, autoimmune thyroid disease; MV, mixed vitiligo; NSV, non-segmental vitiligo; SV, segmental vitiligo; AD, autoimmune disease; Immune serology, immunoglobulin plus complement; Area of involvement, measured by the area of the patient’s palm (single).

An increasing number of researchers have found that many factors associated with vitiligo are also related to the onset of AITD (32, 33); therefore, we reviewed the results of the data for these. Among vitiligo patients, 165 female patients (69%), 197 patients (83%) with NSV, 208 patients (87%) with thyroid nodules or tumors, 114 patients (48%) with negative emotions, 35 patients (15%) with > 5% of the skin affected, and 49 patients (21%) with positive immune serology displayed AITD symptoms. Patients with vitiligo combined family history of AITD were found in 28 cases (12%). Twenty-nine cases (12%) had AITD, who had patients with vitiligo with a history of autoimmune diseases except AITD (**Table 1**).

### 3.2 Predictor selection

**Figure 1:**
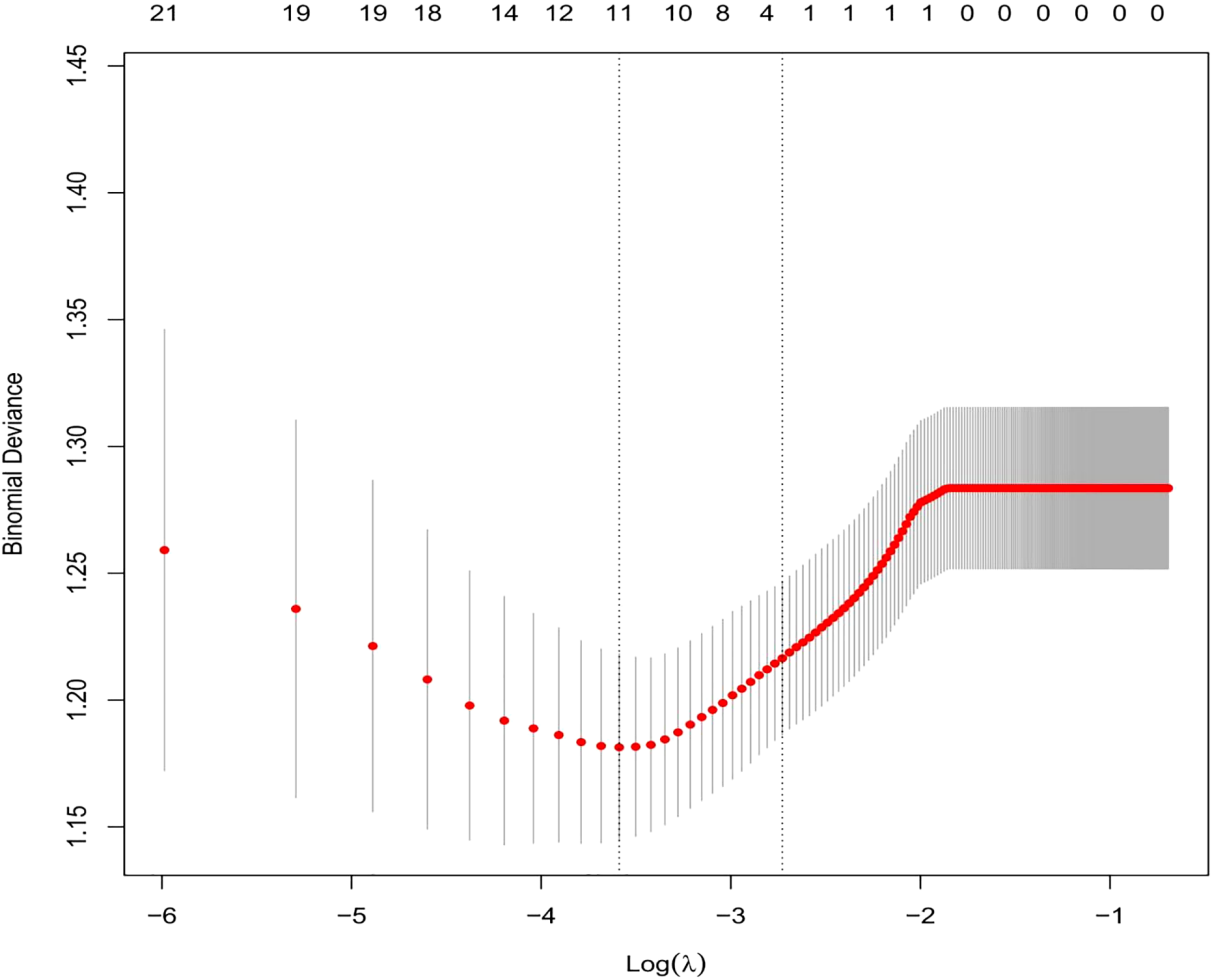
By using the five-weight verification method, the optimal parameter (Lambda) (29) can be selected in the LASSO model. Using partially mild deviation curves (two deviations) and paired graphs (Lambda), a virtual line is drawn vertically at the optimal value to determine the characteristic factor.

**Figure 2:**
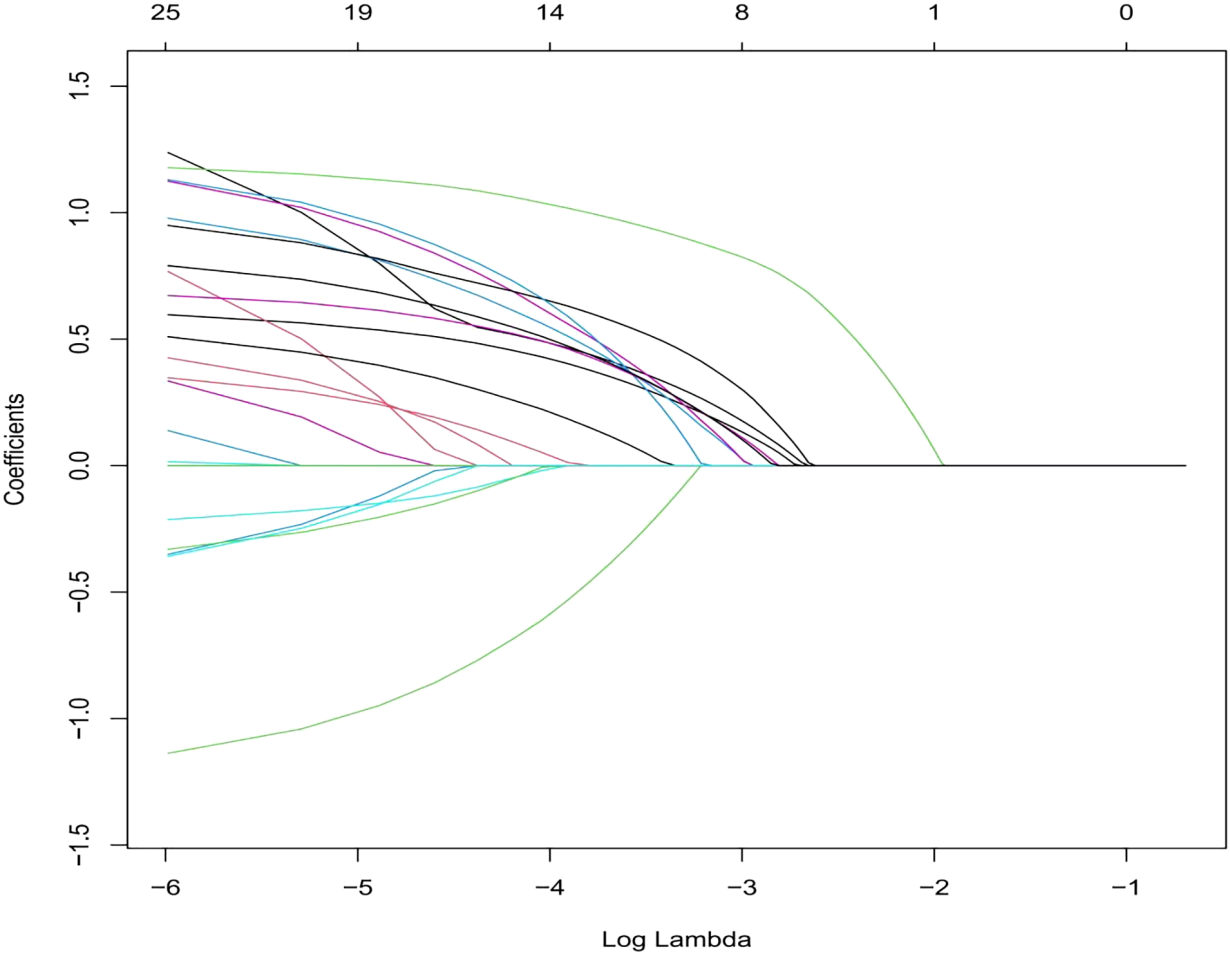
This section consists of 11 characteristics based on the sequence of the number (Lambda) sequence. A vertical line is drawn at the selected value using five weights cross-verification, and the coefficient of five elements with non-zero values is selected.

We selected the following 11 potential predictors in the 703 patients with vitiligo (**Figures 1**, **2**), including sex, age, vitiligo type, family history of AITD, family medical history of non-thyroid autoimmune diseases, thyroid nodules or tumors, PANAS evaluation, smoking history, alcohol history, vitiligo involvement > 5% of body area, and immune serology (immunoglobulins and complement) (**Table 2**).

**Table 2:** Univariate and multivariate logistic regression analyses in autoimmune thyroid disease.

	Multivariate analysis		Univariate analysis	
	Odds ratio (95% CI)	P-Value	Odds ratio (95% CI)	P-Value
Sex	1.85 (1.27-2.73)	0.002	1.78(1.16-2.72)	0.008
Age of vitiligo onset (years)				
≤15	–	–	–	–
>15, ≤30	1.19 (0.41-3.48)	0.75	–	–
>30, ≤45	0.55 (0.14-2.11)	0.38	–	–
>45	0.73 (0.14-3.94)	0.72	–	–
Vitiligo duration (years)				
≤5	–	–	–	–
>5, ≤10	0.77 (0.50-1.17)	0.22	–	–
>10	0.92 (0.34-2.50)	0.86	–	–
Type				
MV	–	–	–	–
NSV	4.23 (1.75-10.24)	<0.001	4.44 (1.67-11.79)	0.003
SV	2.28 (0.87-5.97)	0.09	2.73 (0.95-7.88)	0.063
Family vitiligo history	0.81 (0.41-1.60)	0.55	–	–
Family AITD history	3.51 (1.50-8.19)	0.004	2.34 (0.90-6.11)	0.083
History of ADs except thyroid	1.23 (0.72-2.10)	0.45	–	–
Family history of ADs except thyroid	3.25 (1.38-7.67)	0.007	2.50 (0.93-6.73)	0.07
Thyroid nodules or tumors	3.28 (1.69-6.36)	<0.001	2.94 (1.37-6.29)	0.005
Trace elements	1.23 (0.76-2.00)	0.40	–	–
Mood	3.87 (2.58-5.80)	<0.0001	3.202(2.08-4.93)	<0.001
Hypertension	1.98 (0.28-14.19)	0.50	–	–
Hyperlipidemia	1.32 (0.22-7.96)	0.76	–	–
Blood sugar status	1.32 (0.22-7.96)	0.76	–	–
Cigarette history				
No smoking	–	–	–	–
Smoking	2.01 (1.04-3.89)	0.04	–	–
Quit smoking >6 months	1.36 (0.57-3.22)	0.48	–	–
Alcohol use				
No drinking	–	–	–	–
Quit drinking >1 year	0.96 (0.46-2.02)	0.91	–	–
Drinking <3 times a week	2.23 (0.89-5.61)	0.09	–	–
Drinking >4 times a week	0.67 (0.21-2.11)	0.49	–	–
Area of involvement	2.90 (1.52-5.50)	0.001	2.03(1.00-4.17)	0.051
Immune serology	2.23 (1.34-3.69)	0.002	2.16(1.23-3.80)	0.008

CI, confidence interval; MV, mixed vitiligo; NSV, non-segmental vitiligo; SV, segmental vitiligo; AITD, autoimmune thyroid disease; AD, autoimmune disease; Immune serology, immunoglobulin plus complement; Area of involvement, measured by the area of the patient’s palm (single).

### 3.3 Construction of the personalized prediction model

**Figure 3:**
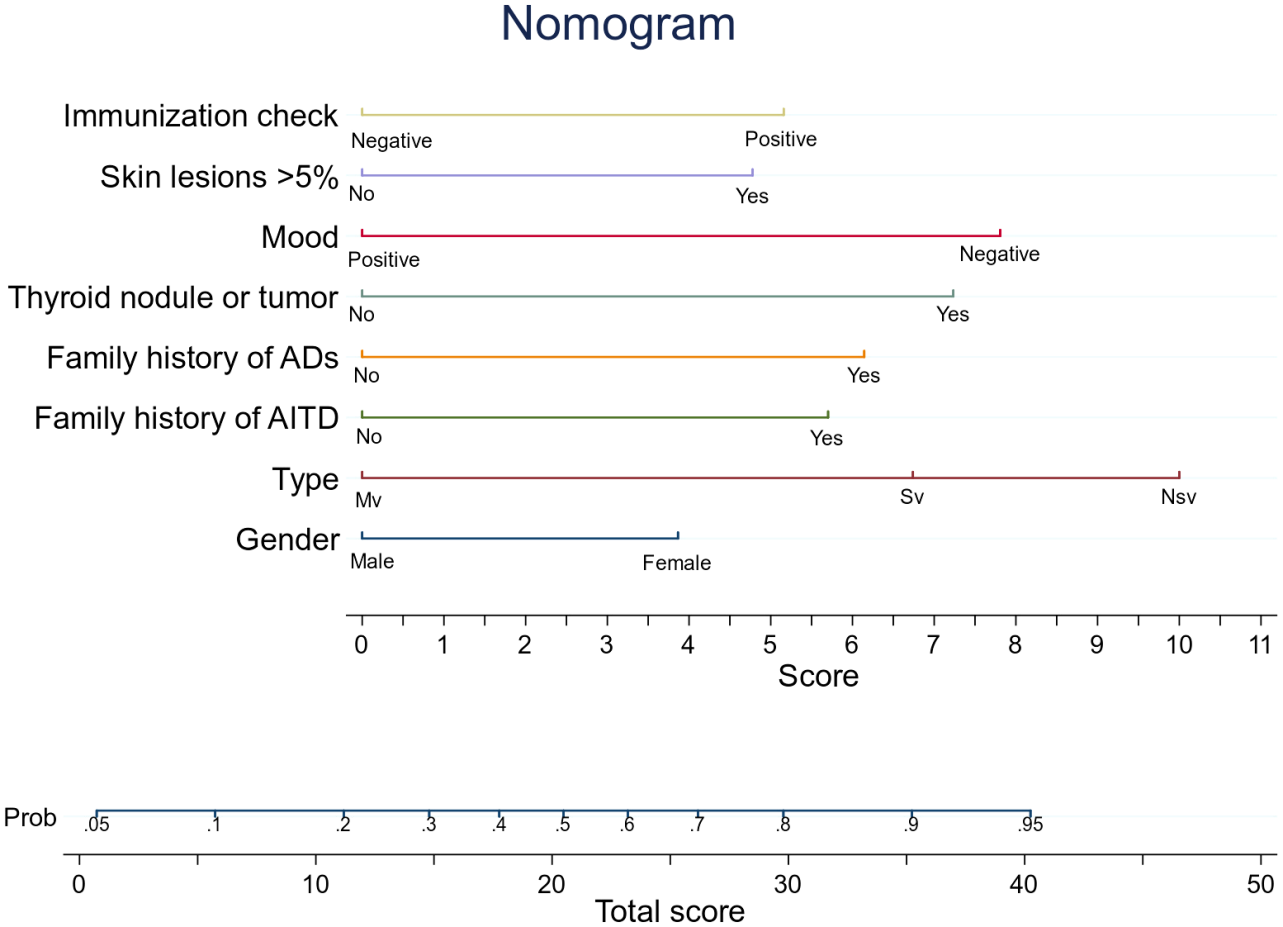
An AITD prediction line diagram may be developed in the queue based on a number of factors, including sex, vitiligo type, AITD family history, except for AITD autoimmune disease family history, thyroid nodules or tumors, and negative emotions. Involved skin area exceeds 5% of body surface area, and immunoglobulin plus tonic is positive. To calculate the probability that patients with vitiligo will develop AITD, draw a straight line on the point axis (for example, patients with non-segmental vitiligo will get 10 points). For each variable, repeat this process. Find the sum of the axis corresponding to each predicted variable. To determine the probability that patients with vitiligo will develop AITD, draw a line directly. For example, if a female patient with vitiligo has a family history of AITD, which is greater than 5%, and has a negative emotional type, then the prediction equation indicates that she has 53% chance of developing AITD. LASSO, Least Absolute Shrinkage and Selection Operator; AITD, autoimmune thyroid disease. The decision curve of the Hosmer–Lemeshow (HL) calibration curve predicts the nomogram, the area under the curve (AUC), and the risk of autoimmune thyroid disease (AITD).

**Table 2** displays the findings of the logistic regression analysis. Patient sex, vitiligo type, family history of AITD, family history of autoimmune diseases other than AITD, thyroid nodules or tumors, emotion type, vitiligo involvement > 5% of body area, and abnormal immune serology (immunoglobulin plus complement) entered the nomogram as model predictors. The prediction equation used for the nomogram was as follows: LogitP = -3.053 + (0.5676 * female sex) + (0.96 * family history of autoimmune diseases except AITD) + (0.850 * family history of AITD) + (0.178 * combination of thyroid nodules or tumors) + (1.164 * negative emotion) + (0.720 * vitiligo involvement > 5% of body area) + (0.769 * abnormal immune serology) + (1.440 * segmental vitiligo). This predictive model is suitable for predicting the risk of AITD in patients with vitiligo based on current clinical characteristics and laboratory tests. The nomogram creates segments of various lengths to predict the disease based on how significant its predictors are in the overall variables (34). **Figure 3** shows how to use it. The prediction process is statistically consistent, while the images are concise and aesthetically pleasing; we believe this will help with effective communication between doctors and patients.

### 3.4 Validation of AITD risk nomogram

**Figure 4:**
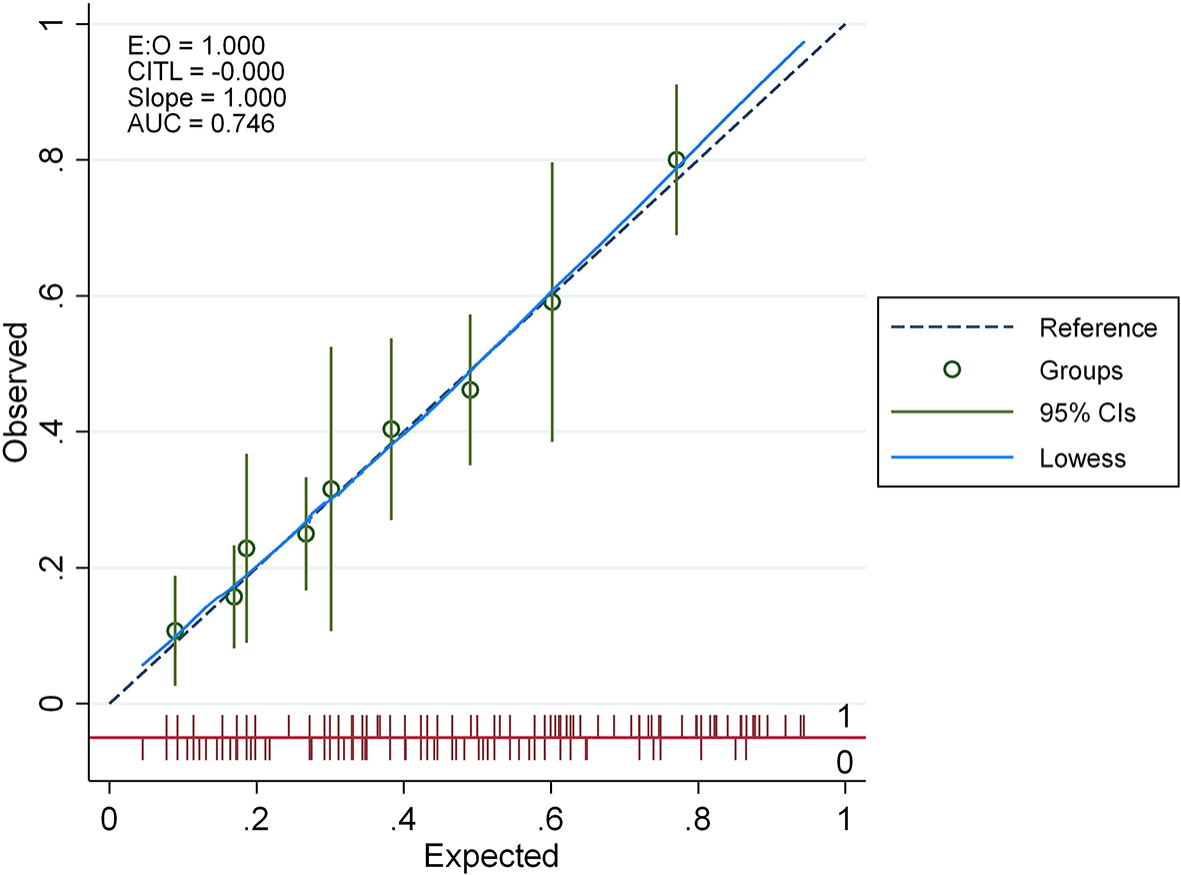
The calibration curve for the AITD prediction nomogram. The expected risk of developing AITD is shown on the x-axis. For the ideal model, diagonal dashed lines reflect accurate predictions. The performance is shown by the solid line, and the more closely it resembles the dashed line, the more accurate the forecasted risk.

**Figure 5:**
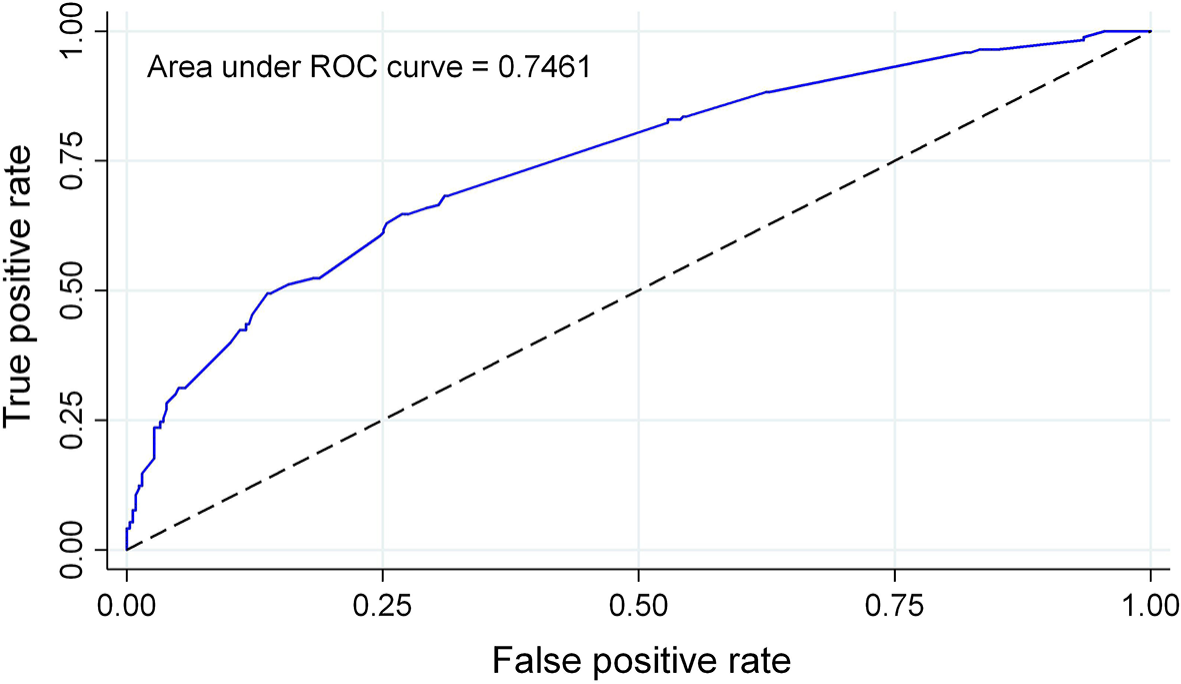
AUC for the AITD prediction nomogram. The probability of correctly predicting whether a patient has AITD in selected random cases is indicated by the AUC of a prediction model. The model has strong predictive power, and the overall sample AUC value is 0.7461.

We developed probability plots for the nomogram and evaluated nomogram calibration for its ability to identify patient outcomes or discriminate patients by comparing the predicted probability with the actual probability plots (35). The calibration curve of the nomogram used to predict AITD risk in patients with vitiligo indicated good agreement (**Figure 4**). The C-index measured the prediction accuracy (discriminant) of the nomogram (35). The accuracy of the predicted nomogram was 0.746 (95% CI: 0.701–0.792), with a bootstrapping validation score of 0.732. At test time, the accuracy was determined to be 0.869 and 0.777 by an external validation cohort; thus, the prediction model showed good discrimination ability. The accuracy of this predictive model was 0.7461 (**Figure 5**), sensitivity was 64.7%, and specificity was 73.1%. The higher the c-index, sensitivity, and specificity, the higher the accuracy of the prediction. In the external validation Group 1, the accuracy of AITD probability, sensitivity, and specificity were 0.869, 72.7%, and 87.1%, respectively. In the external validation Group 2, the accuracy of AITD probability, sensitivity, and specificity were 0.777, 92.3%, and 58.6%, respectively. These results indicated that the AITD predicting model had high predictive ability.

### 3.5 Clinical application

**Figure 6:**
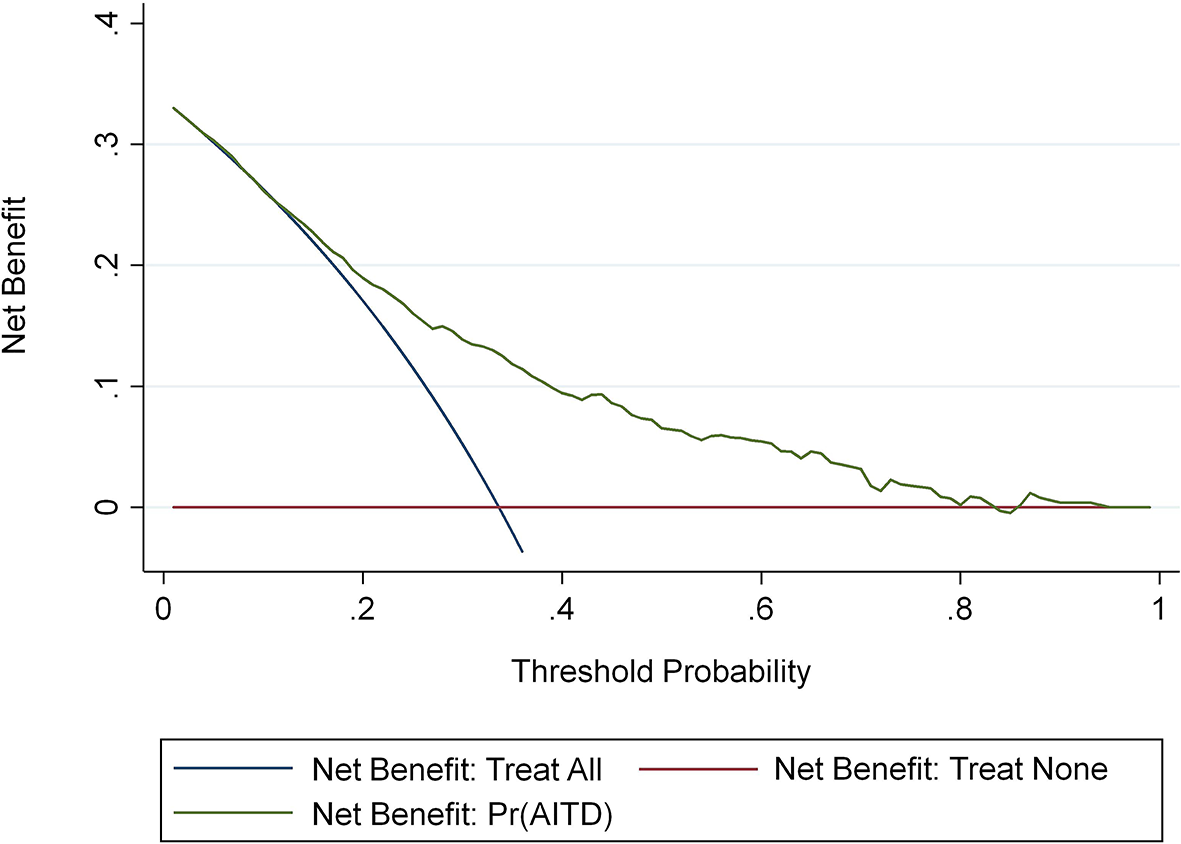
The AITD decision curve to assess the risk of meaning map. The y-axis represents net income. The blue line represents the risk nomogram of AITD. The thin solid line represents the hypothesis that all patients have AITD. The thick solid line represents the hypothesis that no patients have AITD.

We conducted clinical DCA on the nomogram in the training and validation sets to verify the net benefit to patients with vitiligo (36) (**Figure 6**). These results demonstrate that this predictive model is well suited for the prediction of AITD in patients with vitiligo, which can help guide treatment and reduce the risks of missing the AITD diagnosis in patients with vitiligo.

### 3.6 External verification

We also validated two independent external cohorts with the Hosmer–Lemeshow test, receiver operator characteristic curve, and DCA. The results are shown in **Supplementary Material**.

## 4 Discussion

We have found a number of traits that make vitiligo patients more likely to develop AITD, most of which have been mentioned in previous studies. These include Sex, vitiligo type, family history of AITD, family history of other autoimmune disease, thyroid nodules or tumors, negative emotions. Our latest study finds that positive immune serology or skin involvement exceeding 5% of body surface area of the whole were more likely to develop AITD.

One of the most accepted current theories regarding the pathogenesis of vitiligo is an autoimmune origin (11). In addition, the prevalence rate of AITD in patients affected by vitiligo is 14.3% (13). Previous studies have reported that compared to male patients, female patients with vitiligo have a significantly higher probability of having AITD; this is consistent with our findings. Secretion-related (37) androgens (e.g., testosterone) have suppressive effects on immune function. The increased female prevalence may be owing to the immune-boosting effects of estrogenic sex steroids (38). The type of vitiligo is also a risk factor. According to previous studies, AITD is more common in patients with NSV because of its pathogenesis, which differs from SV (25). SV is considered nerve-related, while autoimmune factors are associated with NSV (39). In recent years, SV has also been reported as autoimmune-mediated because of melanocyte-specific T cell infiltration of the same melanin cells as that of NSV (40). At present, we have not tested this theory, and we have to further increase the amount of data to verify it. Epidemiological evidence for genetic susceptibility to AITD has also been confirmed by familial clustering of the disease (22). Approximately 33% of siblings of patients with Hashimoto’s thyroiditis or Graves’ disease also have AITD. This is consistent with the results of our study. Among family members of patients with vitiligo, patients with autoimmune diseases, such as rheumatoid arthritis, psoriasis, and pernicious anemia, were also more likely to have AITD. This may reflect the susceptibility to AITD in family members of patients with vitiligo having autoimmune disease (41). We must also consider that the occurrence of diseases within a family may not only be due to heredity, but also due to external factors such as diet and geography.

Negative emotions are a risk factor for AITD in patients with vitiligo. Although consensus is lacking regarding the pathogenesis of autoimmune diseases, previous studies have concluded that stress indirectly contributes to their course and onset. Improved epidemiological investigations have revealed that patients with Graves’ disease experienced significantly higher levels of life stress than control patients. The impact of stress on Hashimoto’s thyroiditis is still unknown (42) and is of great significance for understanding the development and prognosis of stress diseases. Through its influence on the nervous and endocrine systems, stress directly or indirectly affects the immune system. Through this immune modulation, individuals are more likely to develop autoimmunity, thereby increasing the risk of developing AITD (23).

This study also analyzed blood tests and found that patients with vitiligo combined with a positive immunoglobulin plus complement test are more likely to have AITD. Testing of immunoglobulins included serum IgG, IgM, and IgA, and of complement included C3 and C4. Studies have shown that the C3 and C4 values in patients with vitiligo were significantly lower than those in controls, and reported cases where IgA and IgG concentrations decreased (43, 44). The basic feature of immunoglobulin is that it binds to the corresponding antigen to achieve phagocytosis and dissolution. C3 and C4, as nonspecific proteins, have remarkable characteristics in humoral immunity (20). Patients with vitiligo involvement exceeding 5% of the total body surface area were more likely to develop AITD. We believe that this is related to the vitiligo severity; more severe disease increases the likelihood of an autoimmune thyroid attack. Some studies have suggested that AITD is associated with thyroid cancer; therefore, more attention should be paid to the investigation of a previous history of thyroid nodules or tumors (12).

Some studies said patients with vitiligo combined other autoimmune diseases have been reported to be more likely to develop AITD (12). This was not evident in our study data; this may be related to insufficient patient numbers. In an early model of our predictive nomogram, we considered smoking as a risk factor for AITD in patients with vitiligo (45). However, smoking was excluded when the best model was selected because of the following reasons: we had a higher number of female patients, cultural factor influences, and an inadequately sized smoking population. As a result, we were unable to evaluate this risk.

Our study has several advantages. To our knowledge, this is the first study to develop a nomogram using internal and external validation processes for predicting the risk of AITD development in patients with vitiligo. Previous nomograms created for vitiligo and AITD did not use external validation and thus, lacked credibility. However, our study used external validation. Furthermore, our study analyzed a comparatively large number of patients. This helped in obtaining a wide range of basic patient information. These data can also be used in the future for related studies about vitiligo. We examined the reasons for the occurrence of independent predictors, and added speculation and explanations for some unreported factors, which are yet to be verified and improved by other research teams. Finally, in China, with its large population, the number of patients with vitiligo is substantial with a variable patient economic level. The diagnosis of AITD belongs in the endocrinology department and AITD is a complication of vitiligo. Dermatologists need to have some knowledge of the AITD. There are also limitations to this study. This research is retrospective and is based on an insufficiently diverse population. If possible, patients from different regions and ethnicities should be studied, with external validation. Multicenter validation is also necessary.

## 5 Conclusion

In this study, we developed and validated a practical AITD risk prediction nomogram for patients with vitiligo, based on eight predictors that are relatively easy to obtain. The C-index, Hosmer–Lemeshow test, and DCA results indicated good performance. According to our findings, the onset of AITD in vitiligo patients is closely related with the patient’s sex, type of vitiligo, family history of AITD, family history of autoimmune diseases except AITD, negative emotions, thyroid nodules or tumors, skin patches exceeding 5% of the body surface area, and a positive immune test. Future statistical analysis with samples from random populations will further validate the prediction model, to enhance simple and effective assessment for the risk of AITD. Our study contributes to both reducing the health insurance burden of preventable costs and facilitating communication between physicians and patients.

## Data availability statement

The original contributions presented in the study are included in the article/**Supplementary Material**. Further inquiries can be directed to the corresponding author.

## Ethics statement

This study was approved by the Ethics Committee of Tianjin Academy of Traditional Chinese Medicine Affiliated Hospital, Tianjin, China. This is a retrospective study, approved by the Ethics Committee, without the need to sign an informed consent form. Written informed consent from the patients/participants OR patients/participants legal guardian/next of kin was not required to participate in this study in accordance with the national legislation and the institutional requirements.

## Author contributions

ZM contributed to the research design, data analysis, and manuscript writing. MC, XL, and JL performed data collection and validation. KY conducted clinical diagnosis and sample collection. JZ and TG were clinical specialists and contributed to the manuscript revision. All authors contributed to the article and approved the submitted version.
