# Supplementary material for: Predicting the risk of autoimmune thyroid disease in patients with vitiligo: Development and assessment of a new predictive nomogram
Source: Front Endocrinol (Lausanne). 2023 Jan 31;14:1109925. doi: 10.3389/fendo.2023.1109925 (PMC9927026; doi:10.3389/fendo.2023.1109925)
Supplement: Supplementary file 8 [file Table_2.docx]

R language SCRIPT

1 Data import

library(readxl) data <- read_excel("LASSO fig.xlsx")

View(data)

names(data) [1] "A" "B" "C" "D" "E" "F" "G" "H" "I" "J" "K" "L" "M" "N" "O" "P" "Q" "R" "S" [20] "T" "U" var.X<-names(data)[c(2:6,8:20)] var.X [1] "B" "C" "D" "E" "F" "H" "I" "J" "K" "L" "M" "N" "O" "P" "Q" "R" "S" "T" (form<-as.formula(paste("~",paste0(var.X,collapse = "+")))) ~B + C + D + E + F + H + I + J + K + L + M + N + O + P + Q + R + S + T data[var.X]<-lapply(data[var.X], as.factor)

1.1 The variables in the formula are processed, and the categorical variables are processed as dummy variables

modeldata<-model.matrix(form,data) modeldata2<-as.data.frame(modeldata) modeldata2<-modeldata2[,-1]

View(modeldata2)

1.2 Set up Lasso X and Y

X <- as.matrix(modeldata2) Y <- as.matrix(data$G)

3

library(glmnet)

# The same generates a string lambda

lambdas <- seq(0,0.5, length.out = 200)

# Set a random number

set.seed(123)

# Cross-validate

#alpha = 1 Represents a lasso regression

#nfolds =5 Represents 5-fold cross-validation

cv.lasso <- cv.glmnet(X,Y,alpha = 1,lambda = lambdas,nfolds =5,family="binomial")

pdf("lasso1.pdf",8,8)

plot(cv.lasso)

dev.off() pdf 2

pdf("lasso2.pdf",8,8)

plot(cv.lasso$glmnet.fit, xvar="lambda")

dev.off() pdf 2 cv.lasso$lambda.min [1] 0.02763819 lasso.coef <- coef(cv.lasso$glmnet.fit, s=cv.lasso$lambda.min, exact=F)

write.csv(as.matrix(lasso.coef),"lasso.coef.csv")

bootstrap

library(readxl) data <- read_excel("LASSO 图.xlsx")

#View(data)

library(rms) dd=datadist(data)

options(datadist="dd")

fit<-lrm(G~B+E+H+J+K+M+S+T,data=data,x=TRUE,y=TRUE)

v<-validate(fit, method="boot", B=1000, dxy=T) Dxy = v[rownames(v)=="Dxy", colnames(v)=="index.corrected"] orig_Dxy = v[rownames(v)=="Dxy", colnames(v)=="index.orig"] bias_corrected_c_index <- abs(Dxy)/2+0.5

orig_c_index <- abs(orig_Dxy)/2+0.5

cat(" 原始 C 指数是",orig_c_index,"\n")

c<-rcorrcens(G~predict(fit, newdata=data),data=data)

cat(" 可信区间是",c[1,1]-1.96*c[1,4]/2,"-",c[1,1]+1.96*c[1,4]/2)

STATA SCRIPT

logit Aitd Gender i.Type Family history of AITD Family history of ADs except thyroid Thyroid nodules or tumors Mood Accumulated area immunoglobulin plus complement test if dataset==1

predict prob,pr

roctab Aitd prob if dataset==1

roctab Aitd prob if dataset==0

roctab Aitd prob if dataset==2

roctab Aitd prob if dataset==1,graph

roctab Aitd prob if dataset==0,graph

roctab Aitd prob if dataset==2,graph

pmcalplot prob Aitd if dataset==1,ci

pmcalplot prob Aitd if dataset==0,ci

pmcalplot prob Aitd if dataset==2,ci

dca Aitd prob if dataset==1

dca Aitd prob if dataset==0

dca Aitd prob if dataset==2

logit Aitd Gender i.Type Family history of AITD Family history of ADs except thyroid Thyroid nodules or tumors Mood Accumulated area immunoglobulin plus complement test if dataset==1,or

Nomolog

Spss SCRIPT

The SPSS operating procedures are recorded as follows

Import data, select a data set in the selected case, and then click "Analyze - Region - Binary Logic" to perform single factor logical regression and multi factor logical regression (backward) respectively。The single factor is to bring AITD into the dependent variable，Check HL test and 95% confidence interval and the rest are included in covariates.Then, the factor with p<0.05 is subjected to multi factor variable again.Repeat the last action .Create the optimal model and then use roc dialog box to calculate c index, sensitivity and specificity.Repeat the following for both validation sets.
