# Supplementary material for: Predicting the risk of autoimmune thyroid disease in patients with vitiligo: Development and assessment of a new predictive nomogram
Source: Front Endocrinol (Lausanne). 2023 Jan 31;14:1109925. doi: 10.3389/fendo.2023.1109925 (PMC9927026; doi:10.3389/fendo.2023.1109925)
Supplement: Supplementary file 9 [file Table_3.docx]

|  | A | B | C | D | E |
| --- | --- | --- | --- | --- | --- |
| Attentive | 1 | 2 | 3 | 4 | 5 |
| Active | 1 | 2 | 3 | 4 | 5 |
| Alert | 1 | 2 | 3 | 4 | 5 |
| Excited | 1 | 2 | 3 | 4 | 5 |
| Enthusiastic | 1 | 2 | 3 | 4 | 5 |
| Determined | 1 | 2 | 3 | 4 | 5 |
| Inspired | 1 | 2 | 3 | 4 | 5 |
| Proud | 1 | 2 | 3 | 4 | 5 |
| Interested | 1 | 2 | 3 | 4 | 5 |
| Strong | 1 | 2 | 3 | 4 | 5 |
| Hostile | 1 | 2 | 3 | 4 | 5 |
| Irritable | 1 | 2 | 3 | 4 | 5 |
| Ashamed | 1 | 2 | 3 | 4 | 5 |
| Guilty | 1 | 2 | 3 | 4 | 5 |
| Distressed | 1 | 2 | 3 | 4 | 5 |
| Upset | 1 | 2 | 3 | 4 | 5 |
| Soared | 1 | 2 | 3 | 4 | 5 |
| Afraid | 1 | 2 | 3 | 4 | 5 |
| Jittery | 1 | 2 | 3 | 4 | 5 |
| Nervous | 1 | 2 | 3 | 4 | 5 |

A=this concept applies very little or not at all to the participant

B=this concept applies a little to the participant

C=this concept applies moderately to the participant

D=this concept applies a lot to the participant

E=this concept applies very much to the participant

The first 10 items are positive and the last 10 items are negative, assessing which part scores higher
